# Local and Global Public Health and Emissions from Concentrated Animal Feeding Operations in the USA: A Scoping Review

**DOI:** 10.3390/ijerph21070916

**Published:** 2024-07-13

**Authors:** Elise Pohl, Sang-Ryong Lee

**Affiliations:** 1 Wolfson Institute of Population Health, Queen Mary University of London, Charterhouse Square, London EC1M 6BQ, UK; 2 Aero-Soil Laboratory, Department of Biological and Environmental Science, Dongguk University, Goyang 10326, Republic of Korea

**Keywords:** air pollution, industrial livestock production, global health, occupational health, health impacts

## Abstract

Up to 1.6 million tons of waste is produced annually by each of more than 21,000 concentrated animal feeding operations (CAFOs) located in the United States (USA). These operations give rise to externalities, including adverse local and global health impacts from CAFO waste emissions, which can potentially outweigh their economic viability. However, a shortage of evidence synthesis research exclusively on the impacts of USA-based CAFO waste emissions may hinder effective policy development. This scoping review (ScR) study, adhering to the guidelines from the Joanna Briggs Institute, conducted a search in databases including Scopus, Web of Science, PubMed, and Embase in May 2022, resulting in ten publications that met the inclusion criteria. The results suggest possible exposure of CAFO workers to multidrug-resistant *Staphylococcus aureus* (MDRSA), campylobacteriosis, and cryptosporidiosis. Communities near CAFOs experienced higher rates of adverse health impacts compared to those in non-CAFO areas, with patterns suggesting that proximity may correlate with increased odds of detrimental health effects. Implicit global health threats include methicillin-resistant *Staphylococcus aureus* (MRSA), MDRSA, campylobacteriosis, tuberculosis, and cryptosporidiosis. These studies provide foundational insights into CAFO proximity, density patterns, and adverse public health effects, indicating a need for evidence-informed environmental health policies to minimize local and global risks.

## 1. Introduction

Over the past several decades, increasing demand for animal protein has driven a multitude of shifts in agricultural practices, including the rise of concentrated animal feeding operations (CAFOs) [1,2,3]. These large-scale operations are characterized by confining large numbers of livestock within a facility or feedlot to maximize efficiency [3]. Despite this efficiency, CAFOs have stirred extensive debate over their operational methods and broader implications. To demonstrate this, agribusiness has maintained that this increased efficiency creates scale economies and is a fundamental strategy to end world hunger [4,5]. However, several important factors enable these scale economies and low-cost food products, including taxpayer-funded government subsidies, a paucity of environmental legislation, technical advancements in livestock breeding, and feedstock formulation with pharmaceuticals [3,6,7,8,9]. While efficiency may be maximized, significant external costs to the environment and public health exist, including the challenge of storing and disposing of copious amounts of livestock waste, negating perceived efficiencies [3,10,11]. Although some local environmental damage may be “cleaned up” and paid for by taxpayers, these external public health costs are posited to extend to the local and global public, which we discuss in this work [3,12,13,14,15]. In addition to highlighting findings from evidence included in the scoping review (ScR), we expand on these findings with a discussion offering key themes of the included studies and drawing from Krieger’s ecosocial framework, identifying evidence gaps and making recommendations for future studies. Additionally, tor the purposes of this study, we use the United States Environmental Protection Agency (EPA)’s definition of a CAFO, which confines livestock for at least 45 days a year and holds 1000 beef cattle, 125,000 chickens, 82,000 hens, 2500 swine, or 700 dairy cattle. Additionally, facilities with livestock counts below these are considered to be CAFOs if they release manure and wastewater into any waterway or ditch [16,17].

With more than 21,000 CAFOs in the USA, each can produce up to 1.6 million tons of waste annually [18,19]. Although livestock urine and manure or litter are the main components in CAFO waste, other substances can become waste discretely, such as disinfectants and poultry feathers, or as part of the excretions, including endocrine disruption hormones, heavy metals, nutrients, pharmaceuticals, and pesticides [20,21,22]. Similarly, CAFO waste has several emissions, including greenhouse gases, particularly during decomposition, including ammonia, methane, hydrogen sulfide, nitrous oxide, carbon dioxide, toxic compounds of these gases, volatile organic compounds (VOCs), and bioaerosols. Furthermore, emissions include dust and particulate matter containing dried fecal matter, urine, feed, and dander [20,21].

### 1.1. Local Health Impacts

Research has long highlighted the public health cost of local impacts of CAFO pollution, including considerable degradation of the direct environment and ecosystems, loss of terrestrial and aquatic wildlife and their habitats, and adverse impacts on local populations’ health [23,24,25]. Furthermore, numerous studies have conclusively demonstrated the consequences of occupational exposure to emissions from industrial-raised livestock waste, and critical links have been drawn between waste emissions and adverse health impacts. For example, Donham et al. [26] argued that swine CAFO workers exposed to manure pit gases and particulate matter suffered respiratory ailments, with as many as 70% of exposed workers developing acute bronchitis and 25% developing chronic bronchitis. Furthermore, particulate matter in the air absorbs these gases, VOCs, viruses, and bacteria, eliciting a synergistic effect linked to bronchitis and asthma [26,27,28]. Other related respiratory ailments include acute respiratory distress syndrome (ARDS), chronic sinusitis, occupational asthma, organic dust toxic syndrome (ODTS), and chronic decline in lung function [26,29,30].

In addition to adverse occupational health impacts, proximal communities are exposed to pollutants such as particulate matter, ammonia, and hydrogen sulfide through ambient air [31,32]. To illustrate this, Sneeringer [33] posited that doubling the number of industrially raised swine in an area between 1980 and 2002 was linked to a 6.6% rise in ambient, sulfur-based atmospheric pollution. This heightened exposure can lead to a range of health issues, including respiratory conditions, as residents are exposed to emissions and accompanying odors as they permeate through communities [21,34,35,36]. For example, Merchant et al. [32], Mirabelli et al. [37,38], and Sigurdarson et al. [39] observed links between swine CAFO waste emissions and asthma symptoms in adolescents and children. Similarly, Sneeringer [13] argued that air pollution in a region with a high concentration of swine CAFOs triggered respiratory disorders in infants, leading to a 7.4 percent rise in infant mortality. Similarly, odors emanating from emissions of CAFO waste were suggested to be correlated with symptom patterns, including runny nose, cough, diarrhea, dizziness, headaches, and burning eyes. In addition to the physical symptoms, noxious odors were suggested to adversely impact residents’ mental health and stress levels [36,40,41].

### 1.2. Global Health Impacts

Beyond immediate risks to communities, emissions from CAFOs contribute to larger planetary health degradation, including climate change, land-use changes, and biodiversity loss, precipitating catastrophic implications for global health, including the elimination of human habitats [23,24,42,43]. Furthermore, new weather patterns contribute to increases in infectious, zoonotic, and vector-borne diseases [44,45,46,47,48]. Although biosecurity measures in CAFOs may decrease the risk of development of zoonotic diseases, Leibler et al. [8] (p. 59) argued that confinement operations have their own “ecosystems” that promote zoonosis and disease transmission. To illustrate, a gram of avian influenza (AI)-infected fecal matter carries up to ten billion virus particles; thus, particles from infected feces on workers’ clothing, boots, or equipment may be adequate for transmission, especially if biosecurity and biocontainment measures are neglected [8,49]. Furthermore, air circulation in confinements accelerates particulate movement, driving it into the external environment through exhaust systems. Illustrating this point, Okamatsu et al. [50] posited that inhaling airborne particulates from contaminated feces is what led to Japan’s 2005–2006 AI outbreak [8].

The health implications of emissions are manifold, including the spread of antimicrobial resistance (AMR) as a significant global threat. Although antimicrobials in feedstock enhance livestock growth and prevent disease spread within crowded spaces, studies indicate that nearly 75 percent of the antibiotics are excreted, furthering the development of AMR in waste and the environment [51,52,53,54,55]. For example, Chee-Sanford et al. [52,56], Campagnolo et al. [51], and He et al. [57] found that applying manure on croplands also adds to the spread of AMR bacteria, which may enter the food chain. Furthermore, He et al. [58] suggested that exposure occurs via inhalation and ingestion of particulates with antibiotic-resistant genes, increasing the risk of AMR-related illnesses such as methicillin-resistant *Staphylococcus aureus* (*S. aureus*) (MRSA), which has been linked to serious infections in surgical sites as well as the blood, skin, respiratory system, and urinary tract. Once considered to be a hospital-acquired (HA) condition, since 2000 there has been an increase in community-acquired (CA) MRSA infections [59,60]. This view is also supported by Larsen et al. [60], Carrel et al. [61], and Schinasi et al. [62], as they reported increases in CA-MRSA infections in regions with a higher livestock density, potentially indicating that residents without livestock contact may be at greater risk of MRSA exposure in these regions.

### 1.3. On Conducting a Scoping Review Study

We have highlighted several studies that identified the local and global public health impacts and risks of waste emissions from livestock facilities. However, this evidence is not reflected in policy addressing emissions from CAFO waste. In focusing our study on the USA, we suggest that the continued absence of evidence-based policies in this context may continue to pose significant health threats to both local and global populations through the unsustainability of these operations [3,24,63,64]. The voluntary 2005 Air Compliance Agreement (ACA) made between the EPA under President George W. Bush and agribusiness lobbyists exempted most CAFOs from regulation under the Clean Air Act (CAA) [20,63]. However, nearly two decades later, the 2005 ACA remains controversial and increasingly generates criticism as local, global, and planetary health impacts become even more apparent [24,65,66]. Additionally, the lack of systematic evidence synthesis studies of topical literature focused solely on USA-based CAFOs may stymie the development of important environmental and public health policy within the context of the USA. In order to fill this gap, this study aims to conduct an ScR to scope the relevant literature transparently and systematically for patterns, themes, and gaps, in accordance with the Joanna Briggs Institute’s guidance and the Preferred Reporting Items for Systematic Reviews and Meta-Analysis for Scoping Reviews (PRISMA-ScR) [67,68]. Furthermore, by including only one country in this review, we are able to understand the country-specific patterns more fully.

To avoid repetition in conducting an ScR solely on USA-based CAFOs, databases were searched for relevant evidence synthesis studies. Preliminary searches were performed in the Cochrane Database of Systematic Reviews, JBI Evidence Synthesis, International Prospective Register of Systematic Reviews (PROSPERO), Systematic Reviews for Animals and Food (SYREAF), and PubMed. Systematic reviews by O’Connor et al. [69,70] and Douglas et al. [71] were identified. Although all papers addressed potential associations between health impacts and pollutants from industrial livestock production (ILP), a multinational context approach was taken, including evidence from Europe, Scandinavia, Canada, and the USA. However, the policies that govern these countries’ industrial livestock sectors are not uniform. For example, ILP emissions are strictly regulated in the Netherlands, creating a significant regulatory policy gap between the Netherlands and the USA, where, as noted previously, oversight is limited [42]. Additionally, funding for O’Connor et al. [69,70] included the National Soybean Council and the National Pork Board, key agribusiness stakeholders in the USA. This evidence synthesis study will fill an important gap by systematically scoping peer-reviewed literature on CAFOs that are located in the USA. This ScR aims to synthesize, examine, and map evidence from 2016–2022 on the local and global public health impacts of airborne and atmospheric emissions from USA-based CAFO waste, and to answer the following questions:

What does the evidence from 2016–2022 indicate about the local (occupational and community levels) and global health impacts from USA-based CAFO waste emissions?

- What are the main findings in the included evidence?
- What are the key themes in the included evidence?
- Where are the gaps in the evidence?
- What recommendations can be made for further research?

## 2. Materials and Methods

This ScR followed the Joanna Briggs Institute (JBI)’s methodology guidance and was conducted in accordance with the Preferred Reporting Items for Systematic Reviews and Meta-Analysis Extension for Scoping Reviews (PRISMA-ScR) checklist [67,68].

### 2.1. Inclusion Criteria Using the PCC Framework

The population, concept, and context (PCC) framework for inclusion criteria links the research questions with the evidence screening process [67]. Criteria must be met for a paper to be included in the review.

#### 2.1.1. Population

This review considered studies that included populations at the occupational, community, and global levels. At the occupational level, this study included CAFO workers and farmers. At the community level, this study included residents in the community or zip code of the CAFO. At the global level, the global population was included.

#### 2.1.2. Concept

This ScR considered studies that explored possible health impacts or risks from exposure to CAFO emissions, which may include a medically diagnosed or self-reported health condition, illness, disease, infection, syndrome, symptom, manifestation, or exposure.

#### 2.1.3. Context

This review considered studies in which the CAFO was within the states or territories of the USA and was a CAFO as defined by the EPA (defined in Section 1). The CAFO livestock type may include swine, chickens, hens, beef cattle, or dairy cattle.

### 2.2. Types of Sources

This ScR considered peer-reviewed sources with quantitative, qualitative, and mixed-methods study designs for inclusion. Additionally, experimental studies involving animals or surrogates were considered for inclusion.

### 2.3. Exclusion Criteria

Following the JBI [67] guidance, the exclusion criteria aided in removing ineligible records during the screening process to narrow the scope to the most relevant literature.

- Studies where the full text is unavailable;
- Studies that have not been peer-reviewed;
- Systematic review studies;
- Studies before 2016;
- CAFOs not located in the USA;
- Studies not examining health impacts.

### 2.4. Search Strategy

The search strategy aimed to locate published, peer-reviewed studies. An initial limited search of Scopus and Web of Science was undertaken to identify search terms in relevant articles on the topic. Identified free-text words in the titles and abstracts of relevant articles, along with index terms describing the articles, were used to create the initial comprehensive search strategy. The search strategy (Supplementary Material S1) was peer-reviewed by a thesis supervisor and an academic health sciences librarian, both at Queen Mary University of London. The search strategy was then adapted for each database’s search operators. The search was conducted in four academic databases: PubMed, Scopus, Web of Science, and Embase. The search was narrowed to the English language and the dates 2016–2022 on 19–20 May 2022.

### 2.5. Evidence Selection

Following the search, all identified records were collated and imported into EndNote 20.3 (Clarivate Analytics, PA, USA). After duplicates were removed, the remaining citations and abstracts were imported into the JBI System for the Unified Management, Assessment, and Review of Information (JBI SUMARI; JBI, Adelaide, Australia) [72]. Evidence screening was conducted in two phases: First, following a pilot test, titles and abstracts were screened for study identification against the inclusion criteria. If a paper could not be conclusively eliminated in phase 1, it was kept for phase 2. Potentially relevant full-text papers were obtained for phase 2. Full-text papers that did not meet the inclusion criteria were eliminated. Any uncertainty that evolved regarding inclusion and exclusion was discussed with the thesis supervisor.

### 2.6. Data Extraction

Data were extracted and charted from included papers using a data extraction tool modified from the JBI SUMARI extraction tool to allow for charting as relates to specific research questions and objectives. The data extraction form template is provided in Table S1. The data items extracted included specific details related to each reference for seven items, including concept, context, population, study design, aims, period/duration, and principal findings relevant to the review questions. A pilot test on the extraction form was conducted after two studies to allow for chart modifications, rechecking, validation, and ensuring consistency and that all relevant data were charted.

### 2.7. Synthesis of Results

Studies were grouped by population type. This included occupational health studies, community health studies, or global health studies.

## 3. Results

### 3.1. Study Identification

**Figure 1:**
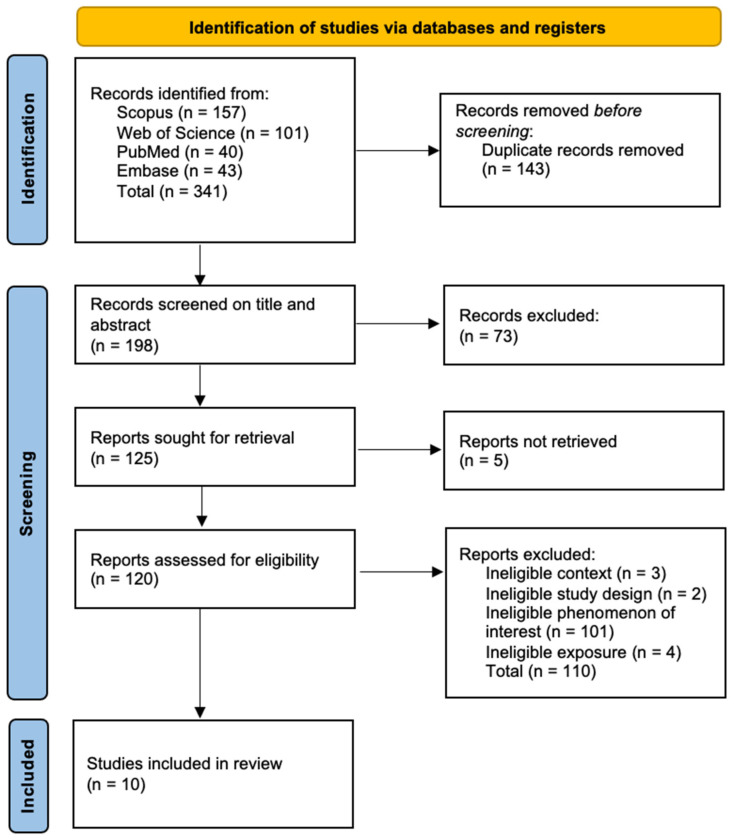
Preferred Reporting Items for Systematic Reviews and Meta-Analysis for Scoping Reviews (PRISMA-ScR) flow diagram [73]. Ten studies met the inclusion criteria of the ScR.

Results were reported on the PRISMA flowchart (Figure 1) [73]. A total of 341 references were identified from four academic database searches. After 143 duplicates were removed, 198 titles and abstracts were imported into JBI SUMARI (JBI, Adelaide, Australia) and screened. During phase 1, 73 references were eliminated. The full text of 125 records was sought for retrieval. Of these, five were not retrieved because they were conference proceedings. The full text of the 120 remaining records was obtained. In phase 2, 110 articles were excluded. Ten papers were included in this ScR. Data were entered into the data extraction tool (Table S2).

### 3.2. Characteristics of Included Studies

**Figure 2:**
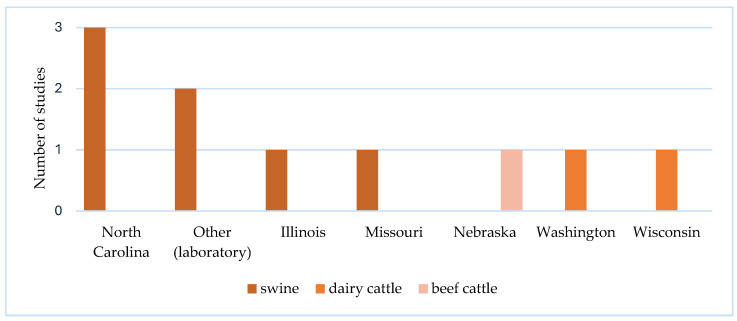
Number of studies by livestock type (*n* = 3), state (*n* = 5), and other (laboratory) (*n* = 2).

The studies were conducted in six different states with three types of livestock (Figure 2). Three studies were conducted in North Carolina (NC). Two studies were conducted in laboratories. One study was conducted in each of the following states: Illinois, Missouri, Nebraska, Washington, and Wisconsin. Five papers were occupational health studies and five were community health studies. Swine CAFOs (*n* = 7) were the most common type of CAFO in the studies. No chicken or hen CAFO studies were identified. Four occupational health studies examined worker exposures in swine CAFOs, and one study analyzed worker exposure in beef cattle CAFOs. Of the five community health studies, two examined exposures in dairy cattle CAFO regions, and three analyzed exposures in swine regions. The study methods include observational study designs (*n* = 7), mixed methods (*n* = 1), qualitative (*n* = 1), and experimental (*n* = 1).

### 3.3. Key Findings of Included Studies

#### 3.3.1. Occupational Health

**Figure 3:**
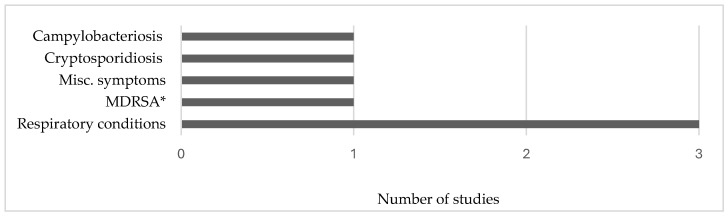
Conditions, exposures, or symptoms investigated in occupational health studies. Miscellaneous symptoms included in Figure 4. Data from Su et al. [74], Davis et al. [75], Knoell et al. [76], Ramos et al. [77], and Wyatt et al. [78]. * Multidrug-resistant *Staphylococcus aureus*.

Five occupational health studies were conducted in Missouri (*n* = 1), Nebraska (*n* = 1), North Carolina (NC) (*n* = 1), and laboratories (*n* = 2). The study methods included qualitative (*n* = 1), experimental (*n* = 1), mixed methods (*n* = 1), and observational (*n* = 2). Five main phenomena of interest were investigated in these five studies (Figure 3). Four studies examined occupational exposures in swine CAFOs.

**Figure 4:**
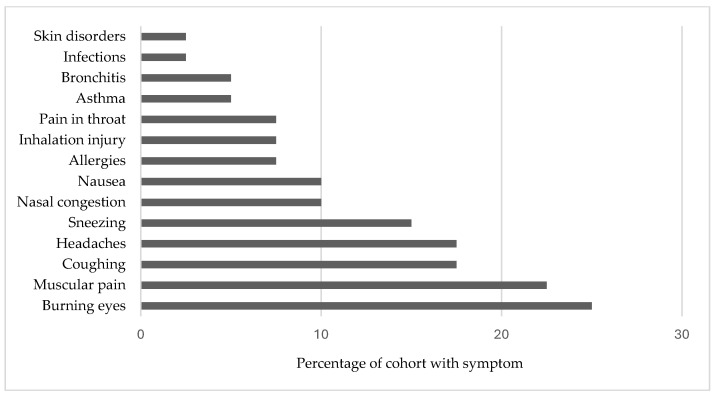
Self-reported condition and symptom data from CAFO workers in Missouri. Data source: Ramos et al. [77].

First, Ramos et al. [77] conducted interviews with 40 Latino immigrant swine CAFO workers in Missouri to collect self-reported data on their health and conditions regarding employment; 42.5% of employees reported poor or fair health, and 28.2% reported ongoing symptoms (Figure 4). The most frequently reported symptoms were burning eyes, muscular pain, coughing, headaches, and sneezing. Additionally, healthcare visits were unaffordable for 30% of workers, who had to postpone healthcare and treatment.

**Figure 5:**
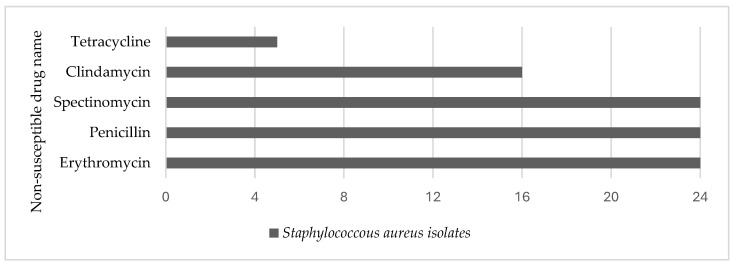
Number of S. aureus-resistant isolates in ambient air at swine facility in NC. Data source: Davis et al. [75].

Next, Davis et al. [75] conducted a pilot test in NC comparing the *Staphylococcus aureus* (*S. aureus*) findings of a swine CAFO that used antibiotics and those of antibiotic-free farms. Utilizing a One Health approach that tested airborne isolates in surrogate workers’ ventilators, on animals, and inside and outside air, they observed that surrogate swine CAFO workers were exposed to *S. aureus* and multidrug-resistant *S. aureus* (MDRSA) through the inhalation of airborne dust, in addition to exposure from swine. Airborne isolate samples collected by Davis et al. [75] at the swine facility were positive for *S. aureus* (Figure 5). All 24 *S. aureus* isolates were multidrug-resistant, belonging to spa type t337, and lacked the scn gene. The isolates were 100% resistant to spectinomycin, penicillin, and erythromycin, but only partially resistant to tetracycline and clindamycin. Conversely, the antibiotic-free farms under comparison identified no *S. aureus* in surrogate workers’ air samplers or in ambient air.

Two laboratory studies were also conducted using swine CAFO dust. Wyatt et al. [78] investigated the influence of alcohol in swine CAFO workers with occupationally induced inflammatory lung disease. They observed that alcohol consumption may modify inflammatory responses in the lungs of swine CAFO workers following exposure to swine dust. Similarly, Knoell et al. [76] investigated whether zinc deficiency impacts the airway inflammatory response to swine CAFO dust. Knoell et al. [76] suggested that zinc deficiency may contribute to the adverse pulmonary function in swine CAFO workers, and that sufficient zinc intake may counter the incidence and severity of swine CAFO dust-induced lung disease.

Lastly, Su et al. [74] investigated occupational animal exposures to campylobacteriosis and cryptosporidiosis in Nebraska using data from the Nebraska Department of Health and Human Services and CDC-reviewed case investigation reports. The highest incidence of campylobacteriosis (*n* = 557) and cryptosporidiosis (*n* = 93) cases reported had occupational exposures at beef cattle CAFOs, with 29.9% and 7.9%, respectively [74].

#### 3.3.2. Community Health Studies

**Figure 6:**
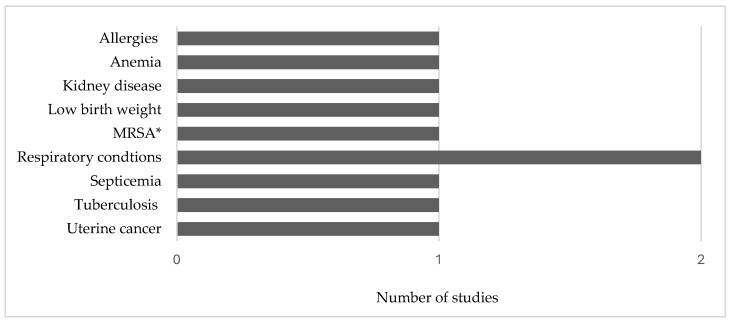
Conditions, symptoms, and exposures investigated in community health studies. Data from Beresin et al. [79], Kravchenko et al. [80,81], Schulz et al. [82], and Loftus et al. [83]. * Methicillin-resistant *Staphylococcus aureus*.

Five community health studies were included in this review. These studies were conducted in Illinois (*n* = 1), North Carolina (*n* = 2), Washington (*n* = 1), and Wisconsin (*n* = 1). All of the studies were observational (*n* = 5). Nine main phenomena of interest were investigated in these five studies (Figure 6).

Three community studies examined adverse health impacts and swine CAFOs. First, in Illinois, Beresin et al. [79] conducted a zip-code-level analysis to investigate methicillin-resistant *Staphylococcus aureus* (MRSA) in skin and soft tissue infections using inpatient hospitalization records. Their research suggested that residents living in zip codes with large farms (>1000 swine) may have higher odds of MRSA infection compared to residents living in zip codes with no CAFOs.

Next, in NC, Kravchenko et al. [80] investigated health outcomes (mortality, hospital admissions, and emergency department visits) of conditions that may be associated with swine CAFOs: kidney disease, anemia, tuberculosis, septicemia, and low birth weight. The investigation compared outcomes in zip codes with swine CAFOs, zip codes with a high density of swine (>215 swine/km), and zip codes with no CAFOs. Secondly, Kravchenko et al. [80] conducted a distance from the source of potential contamination (DiSC) analysis to see if the odds ratios (ORs) changed with proximity to CAFOs. They observed that residents living in areas with zip codes with swine CAFOs and zip codes with high swine density had higher rates of infant mortality, all-cause mortality, multimorbidity mortality, and mortality from tuberculosis, anemia, kidney disease, and septicemia than residents living in zip codes with no swine CAFOs. Additionally, residents in zip codes with swine CAFOs and high swine density had higher rates of hospital admissions and emergency department visits for low birth weight in comparison to zip codes with no swine. Similarly, Kravchenko et al. [80] observed that residents living within 2 km of swine CAFOs had higher ORs of mortality, hospital admissions, and emergency department visits for these conditions than residents living 5 km, 10 km, or 20 km from a CAFO.

Kravchenko et al. [81] investigated race-specific mortality and hospital admissions among White and African-American women with uterine cancer in zip codes with high swine density (>215 swine/km) and no swine CAFOs in NC. DiSC analysis was conducted to investigate whether the ORs changed with proximity to swine CAFOs. Kravchenko et al. [81] observed higher ORs for uterine cancer mortality for White and African-American women living within 2 km of a swine CAFO, in comparison to 5 km, 10 km, or 20 km. After adjusting for cofactors, this was significant for White females but not for African-American women. Additionally, they observed that rates of uterine cancer mortality and hospital admissions were higher for White and African-American women living in zip codes with swine CAFOs than for White and African-American women living in non-CAFO communities.

Another two studies were conducted in dairy cattle CAFO regions. First, Loftus et al. [83] investigated whether exposure to air pollution from clusters of dairy CAFOs in Yakima Valley, Washington was associated with health effects in children with asthma. They observed that children had lower forced expiratory volume in the days following increased emissions, yet no association with asthma symptoms was detected [83]. Schultz et al. [82] investigated asthma, allergies, and lung function in residents and proximity to CAFOs. They observed increased self-reported physician-diagnosed nasal and lung allergies, asthma, usage of asthma medication, and uncontrolled asthma in residents living 1.5 miles from a dairy CAFO in comparison to those living 5 miles from a dairy CAFO [82].

#### 3.3.3. Global Health

Global health risks were identified as implicit findings in the literature. Health conditions examined at the local level and known to be global health threats included tuberculosis, multidrug-resistant *S. aureus* (MDRSA), methicillin-resistant *S. aureus* (MRSA), campylobacteriosis, and cryptosporidiosis.

## 4. Discussion

This review has highlighted important peer-reviewed studies on recent patterns of health impacts from CAFOs in the USA. Adverse health conditions, symptoms, and outcomes experienced by CAFO workers include respiratory conditions, campylobacteriosis and cryptosporidiosis infections, symptoms such as headaches, nausea, muscle pain, and burning eyes, and exposure to *S. aureus* and MDRSA. Conditions and symptoms examined in community-level studies included asthma, nasal and lung allergies, and exposure to MRSA.

Additionally, outcomes for low birth weight, kidney disease, anemia, septicemia, tuberculosis, and uterine cancer were measured and analyzed. Regions with high swine density (>215 swine/km) had higher rates of infant mortality, all-cause mortality, multimorbidity mortality, and mortality from anemia, kidney disease, tuberculosis, and septicemia than regions with no swine CAFOs [80]. Similarly, Beresin et al. [79] suggested that residents living in zip codes with large farms (>1000 swine) may have higher odds of MRSA infection compared to those living in zip codes with no farms. This finding, along with the occupational-level One Health study by Davis et al. [75], meaningfully adds to the literature on understanding indirect and inhalation exposure to resistant *S. aureus,* respectively.

Patterns related to community respiratory diseases correspond with the robust body of knowledge strongly demonstrating the effects of CAFO emissions on community health [32,37,38,84,85]. This includes ammonia emissions, which contribute to creating fine particulate matter when reacting with other atmospheric compounds [86]. Although inhalation exposure links with CAFO workers have already been substantiated, recent studies related to potential community exposure may further help explain the respiratory conditions of residents. Another important pattern in the community-level evidence suggests that closer proximity to a CAFO may be associated with a higher disease burden. For example, Kravchenko et al. [80,81] and Loftus et al. [83] observed that residential proximity to CAFOs may be a significant factor concerning exposure and health impacts. One important pattern noted by Kravchenko et al. [81] suggested that mortality from uterine cancer increased with closer proximity to a swine CAFO in White and African-American women. This pattern is significant because swine manure contains excessive amounts of endocrine-disrupting chemicals (EDCs), which impair the functioning of the endocrine system, potentially impacting reproduction and development, and are also suggested to be linked with uterine cancers [81,87,88]. Furthermore, the inhalation of particulates containing EDCs has more recently been posited as a critical exposure pathway, in addition to ingestion and dermal exposure [89].

### 4.1. Key Themes

To further expound on the findings of this ScR, we draw on Nancy Krieger’s [90,91] ecosocial theory framework of disease distribution to flesh out the themes that emerged in the included literature. These include environmental and occupational health disparities, as well as policy failure. Krieger [92] (p. 223) wrote that “The embodied consequences of societal and ecological context are what manifest as population distribution of inequities in health, disease, and well-being”. Several studies, including those by Ramos et al. [77], Kravchenko et al. [80,81], Su et al. [74], and Loftus et al. [83], observed several disparities impacting vulnerable communities and worker populations. Low-wage and foreign-born populations were impacted by poverty and low education levels, and they faced significant barriers to healthcare access, including language, economic, insurance, distance, and transportation barriers. Moreover, living in areas with high livestock density, as in the studies of Kravchenko et al. [80,81] and Loftus et al. [83], generates further inequities, since high livestock density leads to a greater quantity of waste and, potentially, to more exposures and hazards within CAFO-clustered regions. Although CAFO manure is utilized as a fertilizer on agricultural fields, it is often overapplied within a specific district rather than transported, due to the expense [93,94]. Reduced nitrogen in manure volatilizes as ammonia, polluting both the airshed and groundwater [95,96]. Furthermore, contaminants from livestock excrement will gradually accumulate within living organisms within the ecosystem and continue up the food chain [97]. This bioaccumulation of contaminants among CAFO workers and nearby community residents may substantially impact disease burdens.

Occupational health hazards for livestock agricultural workers have been at the forefront of studies for several decades. Despite having one of the most dangerous professions in the USA, workers in livestock agriculture, many of whom are documented and undocumented foreign-born workers, are often not physically shielded from hazards and lack access to protective equipment and training, making them vulnerable to adverse impacts amidst this multitude of human rights violations [98,99,100]. Ramos et al. [77] asserted that foreign farm workers should be protected under the International Labour Organization (ILO)’s Safety and Health in Agriculture Convention-184 (C-184) [101]. Similarly, CAFO employers are required to provide training in health hazards under the Occupational Health and Safety Administration’s Health Communication Standards, yet oversight is inadequate [77]. Furthermore, farmworkers are excluded from protections by the National Labor Relations Act and the Fair Labor Standards Act, something that Panikkar and Barrett [102] (p. 5) argued may enable employers to be negligent in remuneration and training and “perpetuates structural inequalities”. Given that CAFOs represent a significant economic force in the USA, the financial benefits may be at the expense of the workers and their health and safety when failing to inform and protect employees [77,102].

Apart from the health disparities, policy failure emerged as a theme in the included evidence. Complex policy that is dependent on long-term projections, like public health policy, can result in policy failure if met with a short-sighted perspective [103]. The exemption of the vast majority of CAFO facilities from the Clean Air Act (Section 1.3) may exemplify one of the ways in which special interests are prioritized over the good of the people, resulting in the aforementioned disparities. Furthermore, the ILP sector’s success in gaining exemption from the CAA may be indicative that the rule of law is not applied equally. Agribusiness functions at a level that does not require it to regard the present or future well-being of populations or the planet, nor to inform decisions with transparent evidence. Although evidence substantiates the larger body of literature suggesting associations between CAFO waste and adverse health impacts, the lack of policy creates a narrative around the waste. This narrative conceptualizes manure waste as being confined, local, easily dispersed through land application, and benign. Additionally, in seeking increasing economic efficiency, shorter livestock turnover times, fewer inputs, larger outputs, and higher profits, the agribusiness sector’s financial objectives are aligned with an economic framework that promotes industry regardless of the public health externalities. Furthermore, by advancing biomedical models focused on individual health behaviors as outcome determinants, a population’s realities are obfuscated, leading the populace to believe that they are solely responsible for their own physical, mental, and emotional distress, furthering stigmatization.

### 4.2. Evidence Gaps and Suggestions for Future Research

Several gaps in the literature were identified. A large proportion of the studies excluded from this review focused on the local ecological consequences of CAFO pollution, particularly with a focus on water, while human health problems were indicated but not explicitly investigated. Within the USA, NC, IA, and MN are the states with the greatest numbers of CAFOs, and although three papers were identified in NC, no papers on Iowa or Minnesota were identified. Only one qualitative study with CAFO workers was identified. Studies using surrogates, government data, and experimental studies with animals are important for understanding exposures and outcomes, but a lack of studies conducted directly with CAFO workers may undermine the physical and mental health of CAFO laborers. Primary research, such as participant observation and in-depth qualitative interviews with CAFO workers, can help inform strong labor policy. Longitudinal cohort studies can focus on gathering data regarding the barriers to the use of personal protective equipment, health and safety communication, overall working conditions, and understanding mental and physical health determinants beyond the workplace. Additionally, routine and comprehensive data collection by local health departments can help in assessing occupational health and safety in CAFOs. It is clear from the evidence that CAFO workers need immediate protection through safety training and protective equipment to minimize the risk of respiratory conditions and exposure to infectious diseases that they could potentially pass on to their families and friends, who may further carry these into the community and beyond.

At the community level, the use of routinely collected public health data has been important in understanding patterns as they relate to a geographical area. Further epidemiological studies, such as case–control studies to examine exposures for specific outcomes, will be important for further understanding patterns. For example, a case–control study analogous to that of Kravchenko et al. [80,81] could be conducted in other high-livestock-density regions of the USA, performing both zip-code-level and DiSC analyses. Mixed-methods case–control studies could also be conducted with community residents in high-livestock-density regions, using semi-structured interviews and surveys with case and control groups to understand adverse health impacts more fully. Bioaccumulation of CAFO waste pollution in the ecosystem, including human and non-human animals, may present opportunities for further studies utilizing the One Health approach, as exemplified by Davis et al. [75]. Longitudinal studies with a cohort of residents in areas with high livestock density could also be considered to more accurately collect data related to exposures outside of the community, such as workplace exposures in addition to community exposures. Additionally, focus groups could be helpful in the initial stages of a study to thoroughly understand the physical and mental health concerns of residents, in order to narrow the focus of the study. At the global health level, this ScR identified no studies examining the global health impacts of USA-based CAFOs. Modeling the spread of USA-based CAFO emissions and infectious and zoonotic diseases could be a focus for further exploration.

### 4.3. Limitations and Strengths

The first limitation that we want to discuss is the identification of only ten studies. Although not inclusive of all records in the specified time period outlined in Section 2.4, this study followed the robust methodology of the Joanna Briggs Institute and aligned with the PRISMA-ScR, supporting transparency in a systematic process to scope the evidence, with pre-defined inclusion and exclusion criteria outlined in Section 2.1, Section 2.2 and Section 2.3. These criteria helped to identify the topical literature and pinpoint patterns, gaps, and themes. Another limitation was the use of a single search string to locate academic peer-reviewed research on three levels of public health: occupational, community, and global. This may have been accomplished with more success by employing a less restrictive and more inclusive search string, conducting reference searches, and analyzing grey literature. However, the strength of the search string (S1) is that it was peer-reviewed, modified for each database, and informed by the pre-defined inclusion criteria based on the PCC framework outlined in Section 2.1. The identification of CAFOs in line with the EPA’s standards was another limitation of this study. Researchers do not often record livestock counts, CAFO numbers, or waste storage and disposal plans, usually because this material is not pertinent to their research, nor is it always publicly accessible. Self-identification of CAFOs in a region is often optional, making them difficult to distinguish. Finally, the findings presented in this study may not be applicable to other countries. As previously noted in Section 1, national policies enforcing and regulating industrial livestock pollution are not uniform.

A strength of this ScR is that it was purposively specific to the context of the USA, so as to more fully understand the body of literature within a single nation state, arguing that regulatory policy guiding the ILP industry is not uniform. This work also followed the robust guidance of the Joanna Briggs Institute and PRISMA-ScR. Additionally, the search strategy and pre-defined inclusion and exclusion criteria were informed by the PCC framework. The results highlight peer-reviewed evidence on the potential impacts of CAFO waste pollution, fostering a clearer understanding of the potential impacts within the USA’s context. Furthermore, this paper identified and expounded on key themes, and we identified gaps in the evidence to make recommendations for future research.

## 5. Conclusions

This ScR examined evidence that was diverse in scope but enhanced our understanding of the public health patterns and risks associated with USA-based CAFOs. Ten studies were identified in the review and reported on the PRISMA flowchart. Findings in the evidence indicate patterns and recurrent trends of adverse health impacts in the populations working in CAFOs and those living in communities where there are CAFOs, with data suggesting that distance and density may be contributing factors. Bioaccumulation of pollutants in community ecosystems may also pose significant threats. In addition to synthesizing the findings of the recent literature, a deeper understanding of themes that emerged in the evidence offers a lens into understanding important determinants of disease distribution. In addition to local impacts, implicit global health risks in the literature included tuberculosis, campylobacteriosis, cryptosporidiosis, MDRSA, and MRSA. The patterns identified add to the evidence that emissions may be an important factor (but also one of many determinants) influencing the health of residents and workers.

Multiple evidence gaps and recommendations for further research were identified from this review. Additionally, long-term public and environmental health policy informed by transparent evidence synthesis studies may aid in mitigating the health disparities associated with CAFOs in the USA. A step forward was taken by synthesizing important findings within one nation to examine links between adverse health impacts and CAFOs.

## Data Availability

The original contributions presented in the study are included in the Supplementary Materials, further inquiries can be directed to the corresponding authors.

## Supplementary Materials

The following supporting information can be downloaded at https://www.mdpi.com/article/10.3390/ijerph21070916/s1, Supplementary Material S1: Search strategy and specific modifications for uses in four academic databases, Table S1: Data extraction tool template after modification from the Joanna Briggs Institute SUMARI extraction tool (JBI SUMARI, JBI, Adelaide, Australia), Table S2: Data extraction table with eight categories.
