# Supplementary material for: Local and Global Public Health and Emissions from Concentrated Animal Feeding Operations in the USA: A Scoping Review
Source: Int J Environ Res Public Health. 2024 Jul 13;21(7):916. doi: 10.3390/ijerph21070916 (PMC11276819; doi:10.3390/ijerph21070916)
Supplement: Supplementary file 1 [file ijerph-21-00916-s001.zip › ijerph-3071543-supplementary.pdf]

Supplementary Files

Pohl and Lee (2024)

S1. Search strategy and specific modifications for uses in four academic databases.

Search Strategy

Search string constructed utilizing the PCC (population, concept, context) framework and guidance from Joanna Briggs Institute (JBI, 2021)

2016- 2022; English language

Population: (human* OR occupation* OR worker* OR farm* OR labor* OR labour* OR employee* OR veterinarian* OR public OR population* OR communit* OR neighbor* OR neighbour* OR resident* OR local OR regional* OR planet* OR global)

AND

Concept: (health OR impact* OR consequence* OR hazard* OR illness* OR disease OR morbid* OR mortal* OR outcome* OR sick* OR risk* OR symptom* OR expos*) AND (manure OR feces OR faeces OR fecal OR excre* OR wast* OR pollut* OR bioaerosol* OR emission* OR contamin* OR dust OR particulate* OR particle* OR "climate change" OR "air pollution" OR "global warming")

AND

Context: ("concentrated animal feeding operation" OR "confined animal feeding operation" OR "concentrated livestock feeding operation" OR "confined livestock feeding operation" OR CAFO OR AFO) OR ((concentrated OR confine* OR intensive) AND (animal OR livestock) AND (farm* OR feeding OR agricultur* OR production* OR operation* OR facilit* OR factory OR factories) AND "United States")

Academic database searches: Scopus, Web of Science, PubMed and Embase

Search completed on 19 May- 20 May 2022

Abstract, title and keyword (:ab,ti,kw) (or equivalent title and abstract was used on PubMed database)

Scopus: 157 results

(TITLE-ABS-KEY (({concentrated animal feeding operation} OR {confined animal feeding operation} OR {concentrated livestock feeding operation} OR {confined livestock feeding operation} OR cafo OR afo) OR ((concentrated OR confine* OR intensive) AND (animal OR livestock) AND (farm* OR feeding OR agricultur* OR production* OR operation* OR facilit* OR factory OR factories) AND {United States})) AND TITLE-ABS-KEY ((health OR impact* OR consequence* OR hazard* OR illness* OR disease OR morbid* OR mortal* OR outcome* OR sick* OR risk* OR symptom* OR expos*) AND (manure OR feces OR faeces OR fecal OR excre* OR wast* OR pollut* OR bioaerosol* OR emission* OR contamin* OR dust OR particulate* OR particle* OR {climate change} OR {air pollution} OR {global warming})) AND TITLE-ABS-KEY ((human* OR occupation* OR worker* OR farm* OR labor* OR labour* OR employee* OR veterinarian* OR public OR population* OR communit* OR neighbor* OR

neighbour* OR resident* OR local OR regional* OR planet* OR global))) AND PUBYEAR > 2015 AND PUBYEAR > 2015 (Filter English—)

Web of Science: 101 results

(human* OR occupation* OR worker* OR farm* OR labor* OR labour* OR employee* OR veterinarian* OR public OR population* OR communit* OR neighbor* OR neighbour* OR resident* OR local OR regional* OR planet* OR global) (Topic) and (health OR impact* OR consequence* OR hazard* OR illness* OR disease OR morbid* OR mortal* OR outcome* OR sick* OR risk* OR symptom* OR expos*) AND (manure OR feces OR faeces OR fecal OR excre* OR wast* OR pollut* OR bioaerosol* OR emission* OR contamin* OR dust OR particulate* OR particle* OR "climate change" OR "air pollution" OR "global warming") (Topic) and ("concentrated animal feeding operation" OR "confined animal feeding operation" OR "concentrated livestock feeding operation" OR "confined livestock feeding operation" OR CAFO OR AFO) OR ((concentrated OR confine* OR intensive) AND (animal OR livestock) AND (farm* OR feeding OR agricultur* OR production* OR operation* OR facilit* OR factory OR factories) AND "United States") (Topic) and 2022 or 2021 or 2020 or 2019 or 2018 or 2017 or 2016 (Publication Years) and English (Languages)

Embase: 43 results

#25(human*:ti,ab,kw OR occupation*:ti,ab,kw OR worker*:ti,ab,kw OR farm*:ti,ab,kw OR labor*:ti,ab,kw OR labour*:ti,ab,kw OR employee*:ti,ab,kw OR veterinarian*:ti,ab,kw OR public:ti,ab,kw OR population*:ti,ab,kw OR communit*:ti,ab,kw OR neighbor*:ti,ab,kw OR neighbour*:ti,ab,kw OR resident*:ti,ab,kw OR local:ti,ab,kw OR regional*:ti,ab,kw OR planet*:ti,ab,kw OR global:ti,ab,kw) AND (health:ti,ab,kw OR impact*:ti,ab,kw OR consequence*:ti,ab,kw OR hazard*:ti,ab,kw OR illness*:ti,ab,kw OR disease:ti,ab,kw OR morbid*:ti,ab,kw OR mortal*:ti,ab,kw OR outcome*:ti,ab,kw OR sick*:ti,ab,kw OR risk*:ti,ab,kw OR symptom*:ti,ab,kw OR expos*:ti,ab,kw) AND (manure:ti,ab,kw OR feces:ti,ab,kw OR faeces:ti,ab,kw OR fecal:ti,ab,kw OR excre*:ti,ab,kw OR wast*:ti,ab,kw OR pollut*:ti,ab,kw OR bioaerosol*:ti,ab,kw OR emission*:ti,ab,kw OR contamin*:ti,ab,kw OR dust:ti,ab,kw OR particulate*:ti,ab,kw OR particle*:ti,ab,kw OR 'climate change':ti,ab,kw OR 'air pollution':ti,ab,kw OR 'global warming':ti,ab,kw) AND ('concentrated animal feeding operation':ti,ab,kw OR 'confined animal feeding operation':ti,ab,kw OR 'concentrated livestock feeding operation':ti,ab,kw OR 'confined livestock feeding operation':ti,ab,kw OR cafo:ti,ab,kw OR afo:ti,ab,kw OR ((concentrated:ti,ab,kw OR confine*:ti,ab,kw OR intensive:ti,ab,kw) AND (animal:ti,ab,kw OR livestock:ti,ab,kw) AND (farm*:ti,ab,kw OR feeding:ti,ab,kw OR agricultur*:ti,ab,kw OR production*:ti,ab,kw OR operation*:ti,ab,kw OR facilit*:ti,ab,kw OR factory:ti,ab,kw OR factories:ti,ab,kw) AND 'united states':ti,ab,kw))

#26. #25 AND [english]/lim AND [2016-2022]/py

PubMed: 40 results

(((human*[Title/Abstract] OR occupation*[Title/Abstract] OR worker*[Title/Abstract] OR farm*[Title/Abstract] OR labor*[Title/Abstract] OR labour*[Title/Abstract] OR employee*[Title/Abstract] OR

veterinarian*[Title/Abstract] OR public[Title/Abstract] OR population*[Title/Abstract] OR communit*[Title/Abstract] OR neighbor*[Title/Abstract] OR neighbour*[Title/Abstract] OR resident*[Title/Abstract] OR local[Title/Abstract] OR regional*[Title/Abstract] OR planet*[Title/Abstract] OR global[Title/Abstract])) AND ((health[Title/Abstract] OR impact*[Title/Abstract] OR consequence*[Title/Abstract] OR hazard*[Title/Abstract] OR illness*[Title/Abstract] OR disease[Title/Abstract] OR morbid*[Title/Abstract] OR mortal*[Title/Abstract] OR outcome*[Title/Abstract] OR sick*[Title/Abstract] OR risk*[Title/Abstract] OR symptom*[Title/Abstract] OR expos*[Title/Abstract]) AND (manure[Title/Abstract] OR feces[Title/Abstract] OR faeces[Title/Abstract] OR fecal[Title/Abstract] OR excre*[Title/Abstract] OR wast*[Title/Abstract] OR pollut*[Title/Abstract] OR bioaerosol*[Title/Abstract] OR emission*[Title/Abstract] OR contamin*[Title/Abstract] OR dust[Title/Abstract] OR particulate*[Title/Abstract] OR particle*[Title/Abstract] OR "climate change"[Title/Abstract] OR "air pollution"[Title/Abstract] OR "global warming"[Title/Abstract]))) AND (("concentrated animal feeding operation"[Title/Abstract] OR "confined animal feeding operation"[Title/Abstract] OR "concentrated livestock feeding operation"[Title/Abstract] OR "confined livestock feeding operation"[Title/Abstract] OR CAFO[Title/Abstract] OR AFO[Title/Abstract]) OR ((concentrated[Title/Abstract] OR confine*[Title/Abstract] OR intensive[Title/Abstract]) AND (animal[Title/Abstract] OR livestock[Title/Abstract]) AND (farm*[Title/Abstract] OR feeding[Title/Abstract] OR agricultur*[Title/Abstract] OR production*[Title/Abstract] OR operation*[Title/Abstract] OR facilit*[Title/Abstract] OR factory[Title/Abstract] OR factories[Title/Abstract]) AND "United States"[Title/Abstract])) Filters: English, from 2016/1/1 - 2022/5/20

Table S1. Data extraction tool template after modification from the Joanna Briggs Institute SUMARI extraction tool (JBI SUMARI, JBI, Adelaide, Australia).

Reference	Context	Concept	Population /Sample	Study Design	Aim	Period/ Duration	Principal Findings

Table S2. Data extraction table with eight categories.

Reference	Context	Concept	Population/ Sample	Study Design	Aim	Period/Duration	Principal Findings
Beresin et al., 2017	Illinois, swine, community level	MRSA	Inpatient hospitalizations for SSTI (n = 215,218)	Observational	To examine and understand MRSA's swine-to-community linkages	2008-2011	Statistically significant link between zip-code-level swine exposure and C.A. and H.A. MRSA for inpatient admissions suggests that indirect swine production may elevate MRSA risk.
Davis et al., 2018	North Carolina, swine, occupational level	Staphylococcus Aureus	Worker surrogates (n = 6)	Observational	To identify worker MRSA exposure pathways	120 to 150 minutes/2–2.5 hours	Breathing filters indicated the presence of S. Aureus (7 and 9 CFU/m3, respectively), and S. Aureus isolates were MDRSA (spa type t337).<br>At the grassland facility, by contrast, none of the worker samplers had a positive S. aureus test.
Knoell et al., 2019	Lab, swine, occupational level	Airway inflammation	Veterans in Iowa and Nebraska with 2 years of agriculture experience (n = 123); C57BL mice (n = 6)	Experimental	To determine whether zinc affects HDE-induced airway inflammation	3 weeks	Veterans with COPD who did not consume sufficient zinc had worse pulmonary function.<br>Inhaling hog dust extract triggered an increase in neutrophil and total cell influx, mediator hyperresponsiveness, and intensification of tissue disease. Effects were more pronounced in mice that were zinc-deficient.

Kravchenko et al., 2018	North Carolina, swine, community level	Outcomes for kidney disease, anemia, tuberculosis, septicemia, and low birth weight	Residents with emergency department or hospital admissions; From 221 zip codes with hog CAFO(s): (n ≈ 2,260,000)	Observational	To determine whether poor health outcomes are associated with swine CAFOs beyond demographic, socioeconomic, behavioral, or medical treatment access inequities	2007-2013	All disease-specific mortality rates in regions with >215 swine/km2 were higher than in N.C. and the U.S. Communities near swine CAFOs had increased rates of all-cause, multimorbidity, and neonatal mortality. The infant death rate was as high as 495 per 100,000 for infants less than one year of age. Fifty-six zip codes (n = 400,000 inhabitants) with the top quartile of density at >215swine/km ranked #4 in the U. S. for all-cause death, #1 in the country for mortality from renal illness, #2 for septicemia, and #3 for T.B. as an underlying cause and secondary cause.
Kravchenko et al., 2020	North Carolina, swine, community level	Uterine cancer	Uterine cancer mortality and hospital admissions in 56 southeastern NC zip codes with >215hogs/km2; (n = 1,393,824 person-years)	Observational	To determine how demographic, socioeconomic, behavioral, and medical care access affect uterine cancer mortality and hospitalization	2007-2013	Higher odds of uterine cancer mortality in White females living in southeastern N.C. in zip codes with swine CAFOs than in White women from areas without swine CAFOs.
Loftus et al., 2020	Washington, dairy cattle, community level	Asthma	Children 6-16 with asthma living in Yakima Valley (n = 58)	Observational	To determine whether air pollution may be associated with health impacts in children with asthma	2010-2013	Weakened lung function in children in the days after increased exposure to air pollution. No observed correlation with asthma symptoms.

Ramos et al., 2018	Missouri, swine, occupational level	Occupational health concerns	Latino immigrant CAFO workers, aged 18+, in Audrain, Linn, and Sullivan Counties (n = 40)	Qualitative	Examine self-reported workplace injuries and health concerns	June, July, and August 2015	Size of the facilities varied from 2,800 swine to 80,000 swine. In total, 42.5 percent of employees reported poor or fair health; 28.2 percent experienced occupational health issues such as burning eyes, muscular discomfort, headache, coughing, nausea, nasal congestion, and sneezing. Twelve could not afford to visit a doctor in the previous 12 months, forgoing necessary medical visits. Coughing reports rose as job duration increased (r =.80 and p.01). Being a current smoker was shown to be substantially linked with reporting allergies (r = .57 and p.05).
Schultz et al., 2019	Wisconsin, dairy cattle, community level	Respiratory and allergy	2008-2016 Survey of the Health of Wisconsin respondents, rural residents aged 18+ (n = 5338)	Mixed methods	To evaluate respiratory and allergy health of residents near dairy CAFOs	2008-2016	Compared to persons who lived 5 miles from a CAFO, those who lived 1.5 miles away had a higher incidence of self-reported nasal allergies, lung allergies, asthma, asthma medication, uncontrolled asthma, and having had an asthma attack in the previous 12 months.

Su et al., 2017	Nebraska, beef cattle, occupational level	Campylobacteriosis, cryptosporidiosis	Confirmed/probable campylobacteriosis or cryptosporidiosis cases; 2005–2015, aged 14+ (n = 3,352)	Observational	To estimate animal-related occupational livestock exposures	2005-2015	Exposures in occupational settings occurred in 16.6% of 3,352 campylobacteriosis patients and 8.7% of 1,070 cryptosporidiosis cases. Farming and ranching were the most prevalent (68.2 percent and 78.5 percent, respectively), followed by slaughtering and processing (16.3 percent and 5.4 percent, respectively). Cattle/beef was the most prevalent animal occupational exposure, with exposure to feedlots (concentrated animal feeding operations) recorded in 29.9 percent of campylobacteriosis and 7.9 percent of cryptosporidiosis cases.
Wyatt et al., 2017	Lab, swine, occupational level-Nebraska	Inflammatory lung disease	Human bronchial epithelial cells (BEAS-2B); C57BL mice (n = 6)	Mixed methods	To determine whether alcohol inhibits HDE-induced TNF, ICAM-1, and neutrophil adhesion by inhibiting TNF-converting enzyme (TACE) activity.	8 weeks	Mice that were fed alcohol exhibited reduced airway epithelium ICAM-1 expression, which was triggered by hog dust extract. Lung inflammation induced by hog dust inhalation was associated with increases in TNF, IL-6, IL-8, ICAM-1, and neutrophil adhesion.
